# Involvement of Neuro-Immune Interactions in Pruritus With Special Focus on Receptor Expressions

**DOI:** 10.3389/fmed.2021.627985

**Published:** 2021-02-18

**Authors:** Aylin Ruppenstein, Maren M. Limberg, Karin Loser, Andreas E. Kremer, Bernhard Homey, Ulrike Raap

**Affiliations:** ^1^Division of Experimental Allergy and Immunodermatology, Faculty of Medicine and Health Sciences, University of Oldenburg, Oldenburg, Germany; ^2^Division of Immunology, Faculty of Medicine and Health Sciences, University of Oldenburg, Oldenburg, Germany; ^3^Department of Medicine 1, University Hospital Erlangen and Friedrich-Alexander-University Erlangen-Nürnberg, Erlangen, Germany; ^4^Department of Dermatology, Heinrich-Heine-University of Düsseldorf, Düsseldorf, Germany; ^5^University Clinic of Dermatology and Allergy, Oldenburg Clinic, Oldenburg, Germany

**Keywords:** pruritus, inflammation, neuro-immune, sensory neurons, skin disease, atopic dermatitis (AD), psoriasis, chronic spontaneous urticaria (CSU)

## Abstract

Pruritus is a common, but very challenging symptom with a wide diversity of underlying causes like dermatological, systemic, neurological and psychiatric diseases. In dermatology, pruritus is the most frequent symptom both in its acute and chronic form (over 6 weeks in duration). Treatment of chronic pruritus often remains challenging. Affected patients who suffer from moderate to severe pruritus have a significantly reduced quality of life. The underlying physiology of pruritus is very complex, involving a diverse network of components in the skin including resident cells such as keratinocytes and sensory neurons as well as transiently infiltrating cells such as certain immune cells. Previous research has established that there is a significant crosstalk among the stratum corneum, nerve fibers and various immune cells, such as keratinocytes, T cells, basophils, eosinophils and mast cells. In this regard, interactions between receptors on cutaneous and spinal neurons or on different immune cells play an important role in the processing of signals which are important for the transmission of pruritus. In this review, we discuss the role of various receptors involved in pruritus and inflammation, such as TRPV1 and TRPA1, IL-31RA and OSMR, TSLPR, PAR-2, NK1R, H1R and H4R, MRGPRs as well as TrkA, with a focus on interaction between nerve fibers and different immune cells. Emerging evidence shows that neuro-immune interactions play a pivotal role in mediating pruritus-associated inflammatory skin diseases such as atopic dermatitis, psoriasis or chronic spontaneous urticaria. Targeting these bidirectional neuro-immune interactions and the involved pruritus-specific receptors is likely to contribute to novel insights into the underlying pathogenesis and targeted treatment options of pruritus.

## Introduction

**Figure 1:**
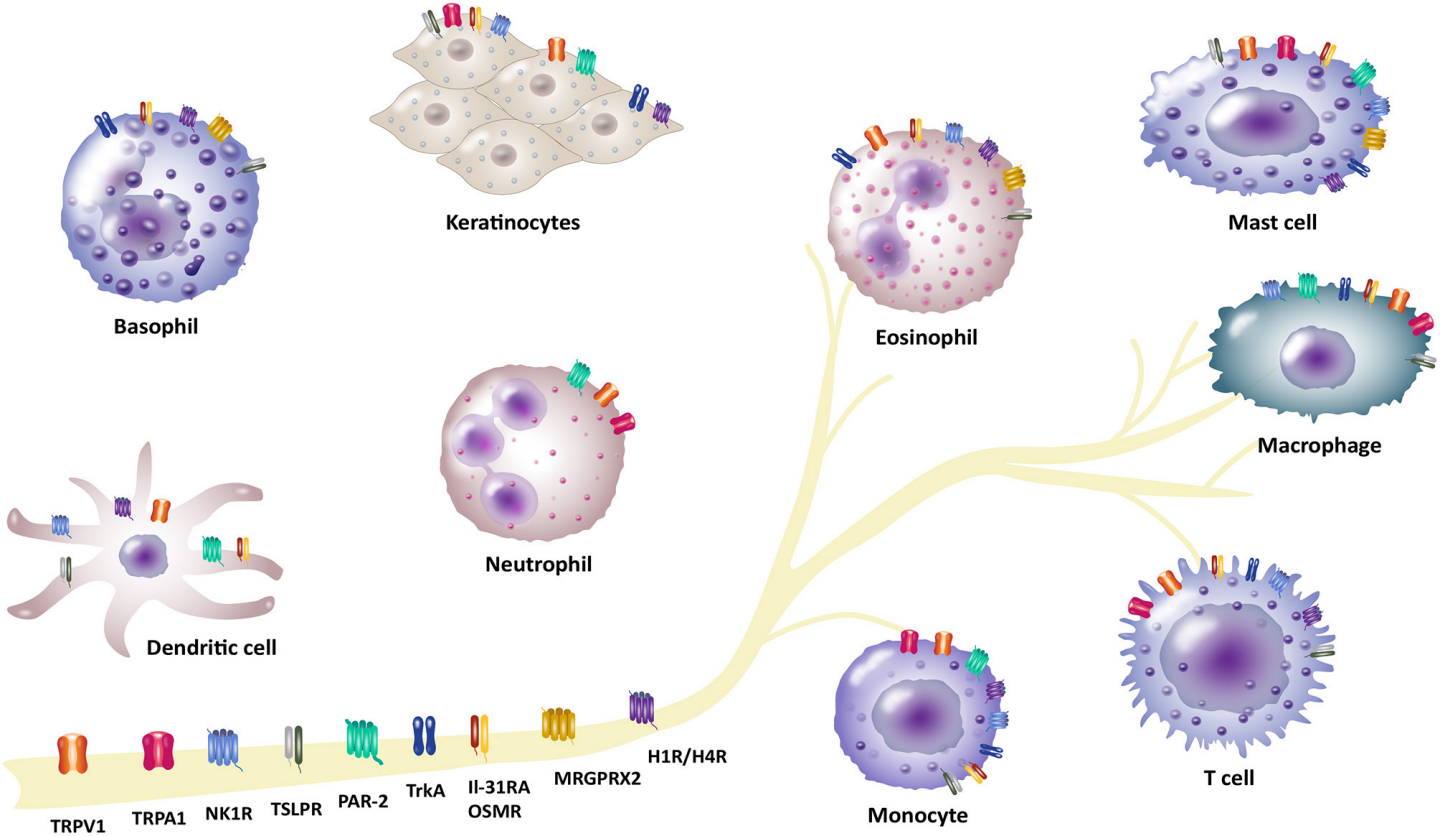
Involvement of different receptors/channels in neuro-immune interactions in pruritus. There is a complex interplay between neurons and immune cells in transmission of pruritus and inflammation. Several receptors act as a bridge between the neuronal and immune network and function as pruritus mediators. These receptors are located on neurons, but also expressed by different non-neuronal cells (e.g., basophils, dendritic cells, eosinophils, keratinocytes, mast cells, macrophages and monocytes, neutrophils or T cells): Transient receptor potential vanilloid 1 (TRPV1) and ankyrin 1 (TRPA1), IL-31 receptor A (IL-31RA) and the oncostatin-M receptor (OSMR), thymic stromal lymphopoietin receptor (TSLPR), protease-activated receptor 2 (PAR-2), neurokinin-1 receptor (NK1R), histamine receptors H1/H4 (H1R/H4R), mas-related G-protein coupled receptor X2 (MRGPRX2), tropomyosin receptor kinase A (TrkA).

The complex symptom of pruritus shows up in several diseases which ranges from numerous inflammatory skin diseases, metabolic disorders, liver and kidney diseases, or lymphoproliferative and myeloproliferative disorders (1). The most common chronic inflammatory skin diseases include atopic dermatitis (AD), psoriasis and chronic spontaneous urticaria (CSU). These patients often suffer from moderate to severe pruritus and experience a reduced quality of life (2, 3). Chronic pruritus in these patients remains a challenge regarding effective anti-pruritic treatments (4). The physiology of pruritus is transmitted by a complex interaction network of cutaneous and neuronal cells (5–7). Thus, it is very important to understand this network and dynamic processes to identify novel signaling pathways and pruritus mediators. Particularly, immune and neuronal systems are not acting separately, but interact rather closely with each other. Neurons modulate the function of immune cells by releasing neurotransmitters and neuropeptides leading to the transmission of pruritus and inflammation. In turn, activation of immune cells leads to the production and release of proinflammatory mediators including several cytokines, chemokines and neuropeptides that trigger neuronal pruritus response and inflammation in the skin (8, 9). These neuro-immune interactions arise not only from an intense biochemical crosstalk between immune cells and neurons, but also from sharing many properties, including receptor and ligand expression, which enables efficient communication between these two systems (10, 11). Thus, linking immune and neuronal systems provides a powerful way to gain insight into complex interactions associated with the neuro-immune interaction mechanism in pruritus. In this review, we highlight recent discoveries and approaches concerning interaction of pruritus receptors and channels in a neuro-immune manner in the field of pruritus research. We set our focus on transient receptor potential (TRP) channels, such as TRP vanilloid 1 (TRPV1) and ankyrin 1 (TRPA1), the heterodimer IL-31 receptor A (IL-31RA) and oncostatin-M receptor (OSMR), thymic stromal lymphopoietin receptor (TSLPR) and different G protein-coupled receptors (GPCR). These GPCRs comprise protease-activated receptor-2 (PAR-2), neurokinin-1 receptor (NK1R), histamine receptors H1 and H4 (H1R/H4R) and mas-related G-protein coupled receptors (MRGPRs) as well as tropomyosin receptor kinase A (TrkA) receptor (Figure 1, Table 1). These receptors and channels have been found on sensory neurons and play a crucial role in pruritus and neuro-immune pathways as well as pruritus associated inflammatory skin diseases (4, 12, 13). Here, we put emphasis on inflammatory skin diseases including AD, psoriasis and CSU that are highly in context with symptom of pruritus. In previous research, several treatment options for patients suffering from these pruritus-associated disorders were described (14–17). Additionally, we outline current therapeutic options in correspondence with these pruritus-associated receptors and channels (Table 2). Targeting neuro-immune pathways may open up new perspectives in terms of the development of more effective pharmacological treatment options for patients suffering from chronic pruritus.

**Table 1:** Expression of receptors/channels on various non-neuronal cells.

**Receptors/channels**	**Non-neuronal cells**	**References**
TRPV1/TRPA1	Dendritic cells (only TRPV1)	(46, 47)
	Eosinophils (only TRPV1)	(48)
	Keratinocytes	(33, 34)
	Macrophages and monocytes	(35–37)
	Mast cells	(39, 40)
	Neutrophils	(41, 42)
	T cells	(25, 43–45)
IL-31RA/OSMR	Basophils	(62)
	Dendritic cells	(68)
	Eosinophils	(61, 64)
	Keratinocytes	(69, 70)
	Macrophages and monocytes	(65–67)
	Mast cells	(13)
	T cells	(25, 57, 71)
TSLPR	Basophils	(89)
	Eosinophils	(90)
	Dendritic cells	(91)
	Keratinocytes	(92)
	Macrophages and monocytes	(94, 95)
	Mast cells	(93)
	T and B cells	(96, 97)
PAR-2	Dendritic cells	(125, 126)
	Keratinocytes	(124)
	Macrophages and monocytes	(125–127)
	Mast cells	(128, 129)
	Neutrophils	(130)
NK1R	Dendritic cells	(148)
	Eosinophils	(149)
	Keratinocytes	(154, 155)
	Macrophages and monocytes	(151)
	Mast cells	(150)
	T and B cells	(152, 153)
H1R/H4R	Basophils	(185)
	Dendritic cells	(186, 187)
	Eosinophils	(13)
	Keratinocytes	(183, 184)
	Monocytes	(187)
	Mast cells	(188)
	T cells	(28, 189–191)
MRGPRX2	Basophils	(206)
	Eosinophils	(206)
	Mast cells	(205)
TrkA	Basophils	(238)
	Eosinophils	(233)
	Keratinocytes	(228)
	Macrophages and monocytes	(239)
	Mast cells	(231, 232)
	T and B cells	(240, 241)

**Table 2:** Emerging therapeutic targets for treatment of pruritus associated inflammatory skin diseases in humans.

**Receptors/channels**	**Therapeutic agents**	**Indications**	**References**
TRPV1/TRPA1	Asivatrep/PAC-14028 (TRPV1 antagonist)	Atopic dermatitis	(54, 55)
IL-31RA/OSMR	Nemolizumab/CIM331 (IL-31RA antagonist)	Atopic dermatitis	(83)
	Vixarelimab/KPL-716 (OSMR antagonist)	Atopic dermatitis	(84)
TSLPR	Tezepelumab/AMG-157/MEDI9929 (anti-TSLP antibody)	Atopic dermatitis	(111)
	Topical spray containing TTLE and GA (TSLP inhibitor)	Atopic dermatitis	(113)
PAR-2	currently not available in humans	–	–
NK1R	Aprepitant (NK1R antagonist)	Atopic dermatitis	(168, 169)
	Serlopitant/VPD-737 (NK1R antagonist)	Atopic dermatitis, Psoriasis	(173)
	Tradipitant/VLY-688 (NK1R antagonist)	Atopic dermatitis	(174)
H1R/H4R	Bilastine (H1R antagonist)	Chronic spontaneous urticaria	(195)
	Adriforant/ZPL-3893787 (H4R antagonist)	Atopic dermatitis	(193)
	JNJ-39758979 (H4R antagonist)	Atopic dermatitis	(202)
MRGPRs	currently not available	–	–
TrkA	Pegcantratinib/CT327 (TrkA inhibitor)	Psoriasis	(255)

## Receptors In Neuro-Immune Interactions

### Transient Receptor Potential Channels TRPV1 and TRPA1

TRP channels are non-selective calcium-permeable cation channels comprising 28 members in mammals that can be categorized in six related protein families including TRPA, TRPC, TRPM, TRPML, TRPP, and TRPV (18, 19). TRP channels are involved in various sensory functions, such as mechanosensation, olfaction, osmolarity, pain, taste and thermoception (20–22). Several studies presented evidence showing that TRPV1 and TRPA1 play crucial roles in pruritus transmission (23–27). TRPA1 is essential in the signaling pathways that promote histamine-independent pruritus (22, 24), whereas TRPV1 is presumed to be required for both histaminergic and non-histaminergic pruritus (23, 28–31). These recent studies used knockout (KO) mice models and corresponding inhibitors and led to the conclusion that TRP channels are necessary in the pruritus pathways initiated by GPCR agonists like chloroquine and histamine (24, 29, 31). On the other hand, Ru et al. (32) demonstrated that TRPV1 and TRPA1 channels are not required for chloroquine activation of nerves by using dorsal skin-nerve preparation of healthy mice. This indicated that these TRP channels could affect other than primary afferent terminals (32). However, both ion channels are well-expressed in primary afferent sensory neurons, but also in non-neuronal cells like keratinocytes (33, 34), monocytes and macrophages (35–38), mast cells (39, 40), neutrophils (41, 42) and T cells (25, 43–45). TRPV1 is additionally expressed in dendritic cells (46, 47) and eosinophils (48). Besides neuro-immune interactions, crosstalk between the channels TRPV1 and TRPA1 and other receptors has been established in previous studies (5, 24, 47, 49, 50). An experimental study of Oh et al. (40) has described a case of complex interactions among nerve fibers and mast cells with the TRPA1 channel. The study demonstrated a neural TRPA1 dependent mechanism comprising interactions between TRPA1+ dermal mast cells and TRPA1+ dermal afferent nerves in a T_H_2-dominated inflammatory environment, which is responsible for the pruritogenesis of chronic pruritus in AD (40). Another example of neuro-immune interactions highlights the involvement of TRPV1 in the crosstalk between neurons and T-lymphocytes (25). Experiments revealed that IL-31 induces pruritus by binding to IL-31RA that is exclusively expressed on TRPV1+/TRPA1+ DRG neurons indicating TRP channels as key mediators of T-cell mediated IL-31-induced pruritus. Interestingly, only around 4% of DRG neurons were observed to be IL-31RA+ (25, 51). Surprisingly, IL-31 was shown to be a potential pruritogen, since injection of IL-31 into the cheek of mice induced profound pruritus, but not pain. This implicates that pruritus and pain may be induced by different subsets of unmyelinated afferents and pruritus specific afferents might exist (25). Interestingly, several studies reported a delayed pruritus after IL-31 injection in mice (52) as well as in patients with AD and healthy volunteers (53). These studies have led to great interest in targeting TRPV1 and developing potential drugs to treat pruritus, especially in AD. In that regard, a topical TRPV1 antagonist termed asivatrep, has shown to significantly improve symptoms (e.g., pruritus, sleep disturbance) of patients with mild-to-moderate AD (54, 55). Further investigations will be needed to unravel the neuro-immune axis involving TRP channels TRPA1 and TRPV1, neurons and different immune cells for new anti-pruritic therapeutic options.

### IL-31 Receptor A and Oncostatin-M Receptor

The novel cytokine IL-31 signals through a heterodimeric receptor composed of IL-31RA and the OSMR. IL-31 is a T_H_2-cell-derived cytokine and the only known ligand for IL-31RA (56–58). It has previously been observed by Cevikbas et al. (25) that T_H_2 cells are main producers of IL-31. Besides T_H_2 cells, other immune cells like basophils, eosinophils or mast cells can produce and release IL-31 (59–62). The IL-31 receptor complex is not only expressed by DRG neurons (63), but also located on non-neuronal cells, such as basophils (62), eosinophils (61, 64), monocytes and macrophages (65–67), mast cells (13), dendritic cells (68), keratinocytes (69, 70), and T cells (25, 57, 71). IL-31/IL-31RA interaction activates signal transduction pathways leading to expression and release of various chemokines, proinflammatory cytokines, regulation of cell proliferation and stimulation of DRG neurons that play important role in pruritus induction and inflammatory diseases (72–74). A number of researchers observed an association between IL-31 and inflammatory skin diseases with severe pruritus including AD (61, 75), bullous pemphigoid (76), cutaneous T-cell lymphoma (77), CSU (78) and psoriasis (79). Regarding treatment approaches, a successful therapy of urticaria using omalizumab led to decreased serum levels of IL-31 (80). Previous research has established a neuro-immune crosstalk between IL-31 receptor, T cells and sensory nerves in pruritus (25). Cevikbas et al. (25) have shown that T_H_2-derived IL-31 is able to activate IL-31RA on TRPV1+/TRPA1+ sensory nerves in the skin causing the pruritus associated with AD. Furthermore, it was shown that the T_H_2-related and atopy-associated cytokine IL-31 directly induces nerve fiber elongation *in vitro* and *in vivo* in mice, suggesting that IL-31-associated nerve fiber elongation could be involved in skin hypersensitivity of AD patients (57). In this regard, IL-31 has been shown to correlate with disease severity and pruritus in AD patients (75). More recent findings have demonstrated that nemolizumab, an anti-IL-31RA antibody that binds to IL-31RA with subsequent inhibition of IL-31 signaling effectively relieves AD-associated pruritus (81, 82). The first clinical study revealed a statistically significant reduced pruritus visual analog scale (VAS) score to about 50% at week 4 compared with 20% with placebo in patients with AD (81). In a subsequent phase II study 264 adults with moderate to severe AD were treated every 4 weeks with nemolizumab in doses of 0.1, 0.5, or 2.0 mg/kg. Treatment led to decrease of pruritus VAS by 43.7% to 63.1% in a dose-dependent manner over a 12-week period compared with a 20.9% decrease with placebo (82). Further, a long-term extension study based on phase II trial resulted in reduced pruritus up to 90%, whereby it was limited by a placebo group (83). Another approach is provided by a human monoclonal antibody KPL-716, which specifically targets the OSMRβ chain and simultaneously inhibits both IL-31 and OSM signaling. Therefore, blocking OSMRβ with KPL-716 may be a potential treatment option of inflammatory skin diseases (e.g., AD) and needs to be clarified in further experiments (84). These studies indicate that IL-31 is an important cytokine for regulating pruritus and AD disease activity.

### Thymic Stromal Lymphopoietin Receptor

TSLP is a four-helix bundle, IL-7-like cytokine, and a member of the IL-2 cytokine family that contributes to the initiation of type-2 inflammation. It is primarily produced by epithelial cells including keratinocytes, fibroblasts and stromal cells, but also by dendritic cells and mast cells (85, 86). TSLP signaling requires a heterodimeric receptor complex that consists of the IL-7 receptor α-chain (IL-7Rα) and the TSLP receptor chain (TSLPR) (87, 88). TSLP receptor is expressed by a variety of cell populations including non-neuronal cells, such as basophils (89), eosinophils (90), dendritic cells (91), keratinocytes (92), mast cells (93), macrophages and monocytes (94, 95), B and T cells (96, 97), but also by neurons (11, 98). The expression of TSLP from these different target cells can be triggered by various stimuli comprising respiratory viruses (99), cigarette smoke extracts (100) as well as several cytokines, such as TNF-α and IL-1β (101). TSLP is known to be involved in various allergic diseases such as AD (102, 103), bronchial asthma (104) and eosinophilic esophagitis (105). There is a growing evidence indicating that TSLP may also play role in other diseases including autoimmune, chronic inflammatory disorders and cancer (86, 106, 107). In terms of AD several studies show that TSLP serum level as well as TSLP level in the skin of AD patients is elevated (102, 103, 108). An overexpression of TSLP in mice models resulted in the development of AD (109, 110). Wilson et al. (98) have demonstrated that intradermal injection of TSLP led to scratching behavior in mice. Additionally, their data confirmed that TSLP released from keratinocytes acts directly on sensory neurons to induce itch-evoked scratching that was depended on TSLPR. Further it was evidenced that both functional TSLPRs and TRPA1 channels are required for TSLP-induced pruritus. A crosstalk between TSLP and PAR-2 was also observed. PAR-2 activation by its agonists SLIGLR and tryptase induced scratching behavior and Ca^2+^-dependent release of TSLP (98). However, the mechanism behind the TSLP-induced pruritus remains to be elucidated in further experimental studies. Targeting TSLP-TSLPR signaling via anti-TSLP therapy like with tezepelumab, a human monoclonal antibody targeting circulating TSLP, might be a promising tool to prevent and treat several diseases associated with elevated TSLP such as AD (111, 112). Contrarily, a phase II clinical trial tezepelumab treatment of patient with moderate to severe AD showed limited efficacy and insignificant pruritus reduction (111). More recently, Fitoussi et al. (113) demonstrated that a topical spray containing *Tambourissa trichophylla* leaf extract (TTLE) and 18β-glycyrrhetinic acid (GA), which inhibits TSLP secretion, efficiently decreases pruritus in AD patients and improves their quality of life.

### Protease-Activated Receptor-2

The PAR family consists of four members, PAR-1, PAR-2, PAR-3, and PAR-4. All together they belong to G-protein coupled receptors activated by proteolytic cleavage of amino-terminal exodomain (114–117). Furthermore, an activation by different proteases generated by endogenous (e.g., proteases from endothelium, epithelium, fibroblast or immune cells) or exogenous sources (e.g., allergens, dust mite and various plants) is possible (118, 119). Existing research recognizes the critical role played by PAR-2 in skin neurogenic inflammation and in pruritic skin diseases such as AD (119–123). PAR-2 is expressed by various cell types including endothelial cells and keratinocytes (124), dendritic cells, monocytes and macrophages (125–127), mast cells (128, 129), neutrophils (130) and sensory nerve fibers (123, 131, 132). Steinhoff et al. (120) reported an increased signaling through PAR-2 that comprises an increased release of endogenous PAR-2 agonist mast cell tryptase followed by a higher occurrence of PAR-2+ nerve fibers in AD patients (120, 133). In addition to the crosstalk between nerve fibers, mast cells and PAR-2, it was shown that PAR-2 synergistically interact with TRPV1 channel resulting in pruritus sensation (134, 135). A key role of TRPV1 channel in PAR2-evoked Ca^2+^ release in differentiated human primary keratinocytes was shown by Gouin et al. (136). They demonstrated that TRPV1 independently regulate the production of inflammatory mediators, such as IL-1β, TNF-α, and TSLP via Ca^2+^ and NF-kB signaling (136). Overexpression of these inflammatory mediators is in connection with inflammatory skin diseases, such as AD or psoriasis (137–141). In a very recent follow-up study Buhl et al. (119) found that PAR-2 regulates neuro-epidermal communication in AD using a mouse model with epidermal overexpression of PAR-2. The research results indicate that PAR-2 signaling in keratinocytes causes epidermal responses leading to neuronal sensory and inflammatory responses in their AD model (119). A promising therapeutic approach presents a PAR-2 pepducin, termed PZ-235. Barr et al. (142) examined the capacity of PZ-235 to suppress skin lesion thickening, inflammation, and pruritus in acute and chronic models of AD. For this, MA-1, a mast cell-degranulating peptide from wasp venom, was utilized to induce severe scratching in mice. Subsequent PZ-235 treatment significantly reduced scratching behavior in mice up to 50%. Further results demonstrated that targeting PAR-2 via PZ-235 application attenuated production of inflammatory factors, leukocyte infiltration, skin thickening as well as severity of skin lesions. Therefore, PZ-235 may have potential in the effective treatment of patients with AD (142). More studies and clinical trials in humans are currently lacking and needs to be investigated.

### Neurokinin-1 Receptor

Neurokinin receptors belong to G protein-coupled receptors and consists of three members, neurokinin-1-3 receptors (NK1-3R) that are implicated in afferent neuronal signal transduction. There are various ligands for these receptors like neurokinin A (NKA), neurokinin B (NKB), neuropeptide K (NPK), neuropeptide-γ (NKγ), endokinin, hemokinin 1 as well as substance P (SP), belonging to tachykinin family, whereas SP binds with high affinity to the NK1R (143–146). Especially, NK1R is known to mainly contribute to transmission of pruritus (4, 12, 147). NK1R is widely expressed by different immune cells, such as dendritic cells (148), eosinophils (149), mast cells (150), macrophages and monocytes (151) and T and B cells (152, 153), but also by keratinocytes (154, 155) and sensory nerve endings (11, 156, 157). Activation of NK1R via SP leads to multiple signaling cascades involving mast cell degranulation and release of proinflammatory mediators, such as histamine, nerve growth factor expression and leukotriene B4 in keratinocytes and neurogenic inflammation resulting in induction of inflammation and pruritus (145, 146, 158). Several studies investigated the role of SP and NK1R in the pathogenesis of pruritus in various diseases like AD, psoriasis and CSU (7, 159–163). Recently, it was reported that SP and its receptor NK1R are overexpressed in pruritic AD and psoriatic lesional skin (164). A previous study demonstrated that increased serum levels of SP in AD patients correlate with pruritus intensity (165, 166). Interestingly, oral treatment with the NK1R antagonist aprepitant led to reduced serum levels of immunoglobulin E (IgE) and SP levels in tissue as well as decreased cutaneous infiltration of regulatory T cells in an NC/Nga mouse model (167). In contrast, clinical studies revealed no significant differences between aprepitant treatment and placebo concerning reduction in pruritus, improvement in pruriginous lesions or quality of life (168, 169). However, another clinical study has shown that the NK1R antagonist serlopitant has potential as a therapeutic agent for the treatment of patients with chronic pruritus by significantly reducing the pruritus symptom (170–172). A phase II clinical study concluded that serlopitant reduced pruritus in patients with mild to moderate psoriasis (173). Another NK1R antagonist, tradipitant, was examined in terms of reduction of pruritus associated with AD through inhibition of SP-mediated itch signaling. Tradipitant treatment improved pruritus and sleep in mild AD (174). Several NK1R antagonists that potentially reduce pruritus activity in dermatological diseases are reviewed by Pojawa-Goła et al. (146) and Reszke et al. (172). Thus, targeting SP and/or NK1R with regard to neuro-immune crosstalk seems to be a promising approach in the treatment of pruritus. In a previous research it was established that Mas-related GPCR X2, which is also activated by SP, induced inflammation (175). Further, it was suggested that SP-induced pruritus may be mediated by MRGPRs rather than NK1R, since SP-induced pruritus was not decreased in *Nk1r* KO mice. Co-injection of QWF and SP in both *Nk1r* KO and wild-type mice led to significantly decreased SP-induced pruritus. Interestingly, an NK1R antagonist termed QWF was shown to have a dual action on MRGPRX2 (176). However, not only the crosstalk between different immune cells, neurons and NK1R, but also the interaction of NK1R with other receptors is an interesting approach for a better understanding of the pathogenesis of pruritic diseases.

### Histamine Receptors H1 and H4

One of the well-characterized pruritogens is histamine. Histamine is released from mast cells and basophils via activation of histamine receptors, which belong to the G protein-coupled receptor superfamily. While four histamine receptor subtypes (H1–H4) exist, notably histamine receptors H1 and H4 are known to modulate pruritus (13, 177–181). Both histamine receptors (H1R and H4R) are extensively expressed in a wide range of cell types involving sensory neurons (182), epithelial cells like keratinocytes (183, 184), but also immune cells, such as basophils (185), dendritic cells (186, 187), eosinophils (13), monocytes (187), mast cells (188) and T cells (28, 189–191). Especially, the H4R is predominantly expressed by immune cells and is in conjunction with lots of functional histamine-mediated inflammatory responses like modulation of cytokine and chemokine release, chemotaxis and cell recruitment as well as upregulation of adhesion molecule expression (192, 193). However, both the H1 and the H4 histamine receptors play pivotal roles in various pruritic skin diseases, such as AD or CSU (188, 194–196). Various H1R antihistamines like ebastine, cetirizine, and levocetirizine were shown to decrease pruritus symptom of patients with CSU by 60–70% (197). A very recent clinical study presented a switch to bilastine, a H1R antagonist, as an optional treatment for patients with CSU, who are unresponsive to H1R antihistamines at the licensed doses (195). Although H1R antihistamines demonstrated convincing anti-pruritic effects in urticaria, they show limited efficiency in other pruritic skin diseases such as AD (11, 197, 198). In the study of Gutzmer et al. (190), it was demonstrated that AD patients express increased levels of H4R on T cells. Upon stimulation of the H4 receptor pruritogenic IL-31 is up-regulated leading to pruritic response (190). H4R antagonists were shown to reduce T_H_2 cytokine production, pruritus and skin inflammation in AD-associated animal models (199, 200). Therefore, new clinical trials using novel H4R antagonists might a promising treatment for patients with AD such as the H4R antagonist JNJ-39758979, which led to an improvement of inflammatory skin lesions in AD patients (193, 201). In addition, marked effects against pruritus in Japanese patients with AD could be observed in a phase II clinical trial, but the development of agranulocytosis by 2 subjects resulted in early trial termination (202). More recently, H4R antagonist adriforant was shown to improve inflammatory skin lesions in patients with AD. Although adriforant treatment cause a 3-point reduction (scale, 1–10) in pruritus, there was no significant difference in comparison to reduced pruritus with placebo (193). Interestingly, a combined treatment of both H1R and H4R antagonists demonstrated an anti-inflammatory effect in an AD mice model that might be a good strategy to treat patients with AD (203).

### Mas-Related G-Protein Coupled Receptors

MRGPRs are G-protein coupled receptors that comprise at least 50 family members in mice, divided into subgroups MRGPRA-H and 8 members in humans named MRGPRX1-X4, D, E, F, and G. Several members of MRGPRs have emerged as critically important receptors in histamine-independent pruritus. They are mainly expressed by sensory neurons and some also by mast cells (5, 8, 204, 205). Recently, human basophils and eosinophils were reported to express MRGPRX2 (206). However, the MRGPRs can be activated by various endogenous and exogenous peptides or molecules, such as antimicrobial host defense or opioid peptides, SP or eosinophilic granules, but also by drugs like vancomycin or chloroquine (CQ) (12, 207). Particularly, MRGPRA3 and MRGPRC11 in mice as well as the human ortholog MRGPRX1 got into the focus of pruritus researchers over the past decade (12, 208). It was shown that CQ activated MRGPRA3 leading to a pruritus signal via the activation of TRPA1 (24). Furthermore, it was demonstrated that expression of MRGPRA3 establishes a subset of nociceptors that specifically mediate pruritus, but not pain in a mouse model. In addition, a deletion of MRGPRA3+ sensory neurons significantly inhibits scratching behavior (209, 210). A recent study by Lee et al. (211) determined that Korean Red Ginseng water extract (KRGE) inhibits CQ-induced pruritus by blocking the MRGPRA3/TRPA1 pathway. Interestingly, KRGE has also anti-pruritic effects on the histamine-dependent H1R/TRPV1 pathway, which might provide a dual anti-pruritic candidate agent for the treatment of pruritus patients (211, 212). Moreover, KRGE treatment significantly decreased hyperplasia and hyperkeratosis in the epidermis, infiltration of inflammatory cells and suppressed the overexpression of cytokines in the AD-like skin lesions of AD mice model (167). MRGPRC11 is in addition to MRGPRA3 co-localized and expressed in a subset of TRPV1+ afferents and mediates pruritus induced by BAM8-22 (24, 209, 213). Liu et al. (204) has proven that activation through MRGPRC11-specific agonist BAM8-22 induces scratching in murine models. In a following clinical study, BAM8-22 triggered pruritus and nociceptive sensations in humans in a histamine-independent manner as topical antihistamine-containing cream did not attenuate scratching behavior (214). This indicates BAM8-22 as an endogenous pruritus mediator and MRGPRX1 antagonists may present potential anti-pruritic therapies. The synthetic peptide SLIGRL was long believed to mediate scratching behavior via the PAR-2. However, intradermal injected SLIGRL caused scratching behavior in PAR-2 KO mice similar to that of wild-type mice. Liu and colleagues (215) proved that the pruritus induction of SLIGRL was mediated by MRGPRC11 while its hyperalgesic mode of action was derived from PAR-2 (2, 215). Furthermore, MRGPRX1 is responsible for neuronal activation and scratching behavior induced by both CQ and BAM8-22 (204, 213, 216). To date, there is a lack of knowledge about the involvement of MRGPRX1 in the pathology in chronic pruritic diseases such as AD and the potential role of MRGPRX1 antagonists in affected patients. Recently, researchers have shown an increased interest in MRGPRX2 in terms of pruriceptive receptor and its involvement in pruritic diseases like AD or psoriasis (164, 176, 217, 218). MRGPRX2 is expressed in mast cells and an activation of MRGPRX2 by peptides such as SP results in mast cell degranulation leading to release of proinflammatory factors as well as modulation of neurogenic inflammation and pruritus (219, 220). Previous research has established that both the percentage of MRGPRX2+ mast cells and MRGPRX2+ skin mast cells of patients with CSU were significantly higher in comparison to non-chronic urticaria subjects. It was further shown that SP-induced histamine release from human skin mast cells through MRGPRX2 contributing to neurogenic inflammation (221). Interestingly, Green et al. (222) found out that SP-mediated inflammatory responses were independent of its canonical receptor NK1R and identified MRGPRX2 and its mouse homolog MRGPRB2 as an important neuro-immune modulator and a potential target for treating inflammatory pain. Involvement in pruritus transmission and anti-pruritic treatment therapies remain elusive and needs to be clarified in further studies (222). In a recent study, increased MRGPRX2 mRNA expression in pruritic skin of patients with AD and psoriasis was demonstrated as well (164). However, research has consistently shown that only few endogenous agonists for most of these receptors are known so far and their role in the pathogenesis chronic pruritus diseases such as AD remains still unclear.

### Tropomyosin Receptor Kinase A

Trk receptors were firstly described in 1986 and three members of the tyrosine kinase receptor family, TrkA, TrkB, and TrkC, have been identified so far. Trk receptors are activated by various neurotrophins including nerve-growth factor (NGF), brain-derived neurotrophic factor (BDNF), neurotrophin-3 (NT-3) and neurotrophin-4 (NT-4) (223–226). The main source of NGF are keratinocytes in the skin (227, 228), but it is also expressed and secreted by other immune cells, such as basophils (229), monocytes and macrophages (230), mast cells (231, 232) and eosinophils (233) as well as by neurons (234, 235) during inflammation. NGF binds with high affinity to its receptor TrkA as well as the low-affinity neurotrophin receptor p75NTR. TrkA is widely expressed across the airway smooth muscles, the lung epithelium and sensory neurons (236, 237), but also located on various non-neuronal cells like basophils (238), eosinophils (233), keratinocytes (228), monocytes and macrophages (239), mast cells (231, 232) as well as B and T cells (240, 241). Both NGF and its receptor TrkA are suggested to play important roles in pruritus and allergic inflammation. Several studies reported that NGF in the skin and NGF serum levels of AD and psoriatic patients as well as serum levels of patients with asthma are increased (227, 242–245). Additionally, an increased TrkA expression in keratinocytes of patients with AD has been observed during inflammation (228). In AD it was shown, that increased peripheral serum levels of BDNF significantly correlate with disease severity and pruritus (246, 247). Also scratching activities were significantly correlated to increased levels of BDNF as shown by Hon and colleagues (248) which used a DigiTrac model to assess scratching activities in children with AD. In this regard, it has been shown that eosinophils are a source of BDNF and release BDNF and are functionally activated by BDNF with induction of chemotaxis (246–248). Thus, the question arises if BDNF which is released by eosinophils of AD patients is also capable to stimulate nerves. This has recently been shown in a study by us in which we could see that BDNF released by peripheral blood eosinophils of patients with AD led to a significant sprouting of peripheral nerves derived from spinal neurons of mice (247). Thus, also BDNF seems to have an important impact in neuro-immune interaction mechanisms and pruritus. However, NGF affects neurite outgrowth and neuronal survival (236, 249). Interestingly, sprouting of itch-sensitive nerve fibers, promoted by increased NGF levels, has been observed in the skin of patients with AD (242) and in AD-associated mice models (250, 251). Since NGF is known to increase cutaneous innervation in AD models and might contribute to the development of chronic pruritus, NGF and its receptor TrkA could be targets for future treatment of pruritus and allergic inflammation in pruritic diseases like AD or psoriasis. A clinical study demonstrated a promising treatment of AD by neutralizing antibodies against NGF that inhibited the development of skin lesions and epidermal innervation as well as scratching behavior in AD mice model (252). In human sensory neurons, NGF up-regulated the expression and sensitivity of TRPV1 channels by activating TrkA (253, 254). Interestingly, TrkA inhibitor CT327 was shown to significantly reduce chronic pruritus in patients with psoriasis as measured by VAS in a phase II clinical study. The results demonstrated that 62, 46, and 61% of patients treated with CT327 0.05, 0.1, and 0.5%, respectively, had at least a 50% decrease in pruritus VAS in comparison to 32% on vehicle (255). Further trials are necessary to prove the anti-pruritic effects of CT327 in AD. There is growing evidence that cutaneous NGF-TrkA-TRPV1 signaling might be a key mechanism contributing to neurogenic inflammation and pruritus in different dermatological diseases (147, 255).

## Conclusion

Our understanding of the pathogenesis of pruritus has significantly evolved in recent years. There is a growing body of literature on the complex crosstalk between neuronal and immune cells that are involved in the development of acute and chronic pruritus. Neurons directly communicate with and regulate the function of various immune cells, such as mast cells, dendritic cells, eosinophils and T cells in pruritus transmission and inflammation. Immune cells release proinflammatory mediators including cytokines, chemokines, neurotrophins, and neuropeptides that activate sensory neurons to mediate pruritus. Activation of these neurons leads to a release of neurotransmitters and neuropeptides that vice versa have a direct impact on the functional activity of immune cells. The literature on neuro-immune crosstalk has emphasized several key mediators and neuronal pathways involved in the transmission of pruritus. Potential mediator and promising receptor therapeutic targets in the skin as well as in peripheral nerves comprises TRPV1, TRPA1, IL-31RA, TSLPR, PAR-2, NK1R, H1R and H4R, MRGPRs and TrkA, which are highlighted in this review (Figure 1, Tables 1, 2). Future studies targeting neuro-immune interactions will help to unravel the underlying mechanisms of pruritus and to develop specific therapies.

## Author Contributions

AR has performed the literature research and wrote the manuscript including the tables. MML designed the figure and revised the manuscript. UR, KL, AEK, and BH critically revised the manuscript. All authors read and approved the final version of the submitted manuscript.

## Conflict of Interest

The authors declare that the research was conducted in the absence of any commercial or financial relationships that could be construed as a potential conflict of interest.
